# Quest for Anti-SARS-CoV-2 antiviral therapeutics: *in-silico* and *in-vitro* analysis of edible mushroom- *Cordyceps militaris*

**DOI:** 10.1016/j.jaim.2024.100979

**Published:** 2024-06-12

**Authors:** Pradeep Gandhale, Rupesh Chikhale, Pukar Khanal, Vashkar Biswa, Raju Ali, Mohd Shahnawaz Khan, Nilambari Gurav, Muniappan Ayyanar, Sandeep Das, Shailendra Gurav

**Affiliations:** a ICAR-National Institute of High-Security Animal Diseases, Bhopal, Madhya Pradesh- 462 021, India; b UCL School of Pharmacy, 29−39 Brunswick Square, London, WC1N 1AX, UK; c Department of Pharmacology, KLE College of Pharmacy Belagavi, KLE Academy of Higher Education and Research (KAHER) Belagavi- 590010, India; d Department of Biotechnology, Bodoland University, Assam, 783 370, India; e Department of Biochemistry, College of Science, King Saud University, Riyadh, Saudi Arabia; f Department of Pharmacognosy, PES's Rajaram and Tarabai Bandekar College of Pharmacy, Ponda, Goa-403 401, India; g Department of Botany, A.V.V.M. Sri Pushpam College (Autonomous), Poondi (Affiliated to Bharathidasan University), Thanjavur, Tamil Nadu, India; h Department of Pharmacognosy, Goa College of Pharmacy, Goa University, Goa- 403 001, India

**Keywords:** Covid-19, Molecular docking, Molecular dynamics, Network pharmacology, Edible mushroom

## Abstract

**Background:**

The emergence and evolution of SARS-CoV-2 resulted a severe threat to public health globally. Due to the lack of an effective vaccine with durable immunity, the disease transited into the endemic phase, necessitating potent antiviral therapy including a scientific basis for current traditional herbal medicine.

**Objective:**

This study aimed to conduct a pharmacoinformatic analysis of selected chemical ingredients and *in-vitro* evaluation of *Cordyceps militaris* extract against SARS-CoV-2.

**Materials and methods:**

*C*. *militaris*, the widely used fungus in conventional herbal medicine, was subjected to computational investigation using molecular docking, molecular dynamic simulation and network pharmacology analysis followed by the *in-vitro* assay for evaluating its anti-SARS-CoV-2 potential.

**Results:**

The molecular docking analysis of *C. militaris* revealed the Cordycepin's highest affinity (−9.71 kcal/mol) than other molecules, i.e., Cicadapeptin-I, Cicadapeptin-II, Cordycerebroside-B, and N-Acetyl galactosamine to the receptor binding domain of the SARS-CoV-2 spike protein. *C. militaris* aqueous extract could reduce the SARS-CoV-2 viral copy numbers by 50.24% using crude extract at 100 μg/mL concentration.

**Conclusion:**

These findings suggest that *C. militaris* has promising anti-SARS-CoV-2 activity and may be explored as traditional medicine for managing the COVID-19 surge in the endemic phase.

## Highlights

• *Cordyceps militaris* (CM) is the widely used fungus in traditional herbal medicine.
• Docking, MD and network pharmacology of demonstrated antiviral potential.
• MM-PBSA calculations and network pharmacology identified key bioactives.
• CM confirmed as a potent anti-SARS-CoV-2 candidate for the management of COVID-19.

## 1 Introduction

Four years after its emergence as multiple severe pneumonia cases in Wuhan, China, in 2019, the severe acute respiratory syndrome coronavirus-2 (SARS-CoV-2) still represents a significant global public health threat. Owing to its continuous evolution and rapid spread, till April 30, 2024, there were 7,75,335,916 confirmed cases of COVID-19, with 7,045,569 deaths across the continents (www.covid19.who.int). Even though, as of April 30, 2024, a total of 13.59 billion vaccine doses have been administered worldwide, it still poses a significant challenge to the healthcare system, as evidenced by the recent surge of COVID-19 cases globally [1]. Although COVID-19 is still a pandemic, it may eventually turn endemic. The observed evolution and resurgence of SARS-CoV-2 indicates that the virus is unlikely to go extinct anytime soon in the absence of a vaccine that can provide durable immunity. Therefore, it is likely that the virus will eventually move to an endemic phase, at which point new cases of the infection will arise [2]. Furthermore, the endemicity could result in a new pandemic of long COVID that would significantly affect economies and healthcare systems across the countries due to the healthcare facilities required to treat people [3]. Moreover, many traditional herbal therapies for the management of COVID-19 have been reported but the scientific evidence for their effectiveness is pertinent [4,5]. The traditional healers of Sikkim, India, recommend *Cordyceps sinensis* as a tonic [6].

According to *Ayurveda*, cordyceps is a *Rasayana* herb that revitalizes *Prana*. By providing nourishment to the reproductive and adrenal glands, it aids the endocrine system. The production of *shukra*, which include reproductive fluids like vaginal and semen, and the restoration of impaired sexual function are additional functions it performs [7].

Many novel and potentially repurposed compounds are being investigated to find a viable candidate for anti-SARS-CoV-2 medication, but none are deemed compelling enough. The severe and concerning circumstances underscore the pressing requirement for cost-effective and efficient antiviral treatments to combat the disease in resurgence or endemic issues.

Historically, people have valued medicinal fungi for their role in maintaining health and well-being. *Ascomycetes* fungus of the family *Cordycipitaceae* in the genus *Cordyceps* is highly prized, notably in traditional Chinese medicine, due to its wide range of health benefits and great edibility [8]. Most *Cordyceps* species are endo-parasitoids, typically of insect or arthropod hosts [9]. The *Ophiocordyceps sinensis* (formerly known as *C. sinensis*) and *Cordyceps militaris*, both entomopathogenic fungi, are routinely used for their anti-inflammatory, immunomodulatory, lung-improving, and antiviral properties in traditional Chinese medicine. *C. militaris* (L.) Fr. is the most well-known member of the genus, and it resembles a cross between a worm and a grass due to its two main components, the sclerotium (the insect's corpse) and the stalk (the grass part, also known as the fruit body). Fruiting bodies of the fungus are club-shaped and orange in color and develop from the pupae that have died underground. *C. militaris*, also known as orange caterpillar fungi, can be found all over East Asia and has a higher cordycepin content than *O. sinensis*. Cordycepin, its bioactive secondary metabolite, is 3′-deoxyadenosine [10] and exhibits antibacterial, anticancer, antiviral, and antifungal properties. Cordycepin has been found to inhibit protein kinase activity, decrease cell proliferation, and block RNA and DNA synthesis [11]. The immunomodulatory, hypoglycemic, and antioxidant effects of *C. militaris* are attributed to the presence of different polysaccharides and ergosterol. Cordymin, a peptide, arrests the growth of MCF-7 breast cancer cells and exhibits antifungal action. Moreover, *C. militaris* is a good source of fats such as linoleic, palmitic, and stearic acid, as well as amino acids, fatty acids, fiber, ash, proteins, carbs, and trace minerals [12]. The Cordycepin was reported to have antiviral activity against several viruses, including, e.g., adenoviruses, dengue virus, Epstein-Barr virus, hepatitis B virus, hepatitis C virus, Herpes simplex virus 1 and 2, human immunodeficiency virus, human poliovirus, human rhinovirus, influenza viruses, Kyasanur Forest disease virus, Omsk hemorrhagic fever virus, Powassan virus, rotaviruses, vaccinia virus, West Nile virus, yellow fever virus and Zika virus. Moreover, the natural adenosine analogue of fungal origin, Cordycepin (3′-deoxyadenosine), has been found to inhibit the SARS-CoV-2 virus *in-vitro* and time-dependent. Recently, several *in-silico* studies have reported plausible anti-SARS-CoV-2 potential of *C. militaris*. However, most studies focused on *in-silico* studies, and very few were supported by *in-vitro* experimental validation [[13], [14], [15], [16], [17], [18], [19], [20], [21], [22], [23]]. Our proposed research focused on the detailed pharmacoinformatic analysis of crude *C. militaris* extract against SARS-CoV-2 supported by an *in-vitro* study to provide the scientific basis for traditional medicinal purposes. The World Health Organization also emphasizes using scientifically proven traditional medicines to manage COVID-19 [[24], [25]]. This necessitated the evaluation of *C. militaris* for antiviral properties that might provide a scientific basis to use in combating the unpredictable surge of COVID-19 during the endemic phase.

## 2 Materials and methods

### 2.1 Morphological and molecular identification of *C. militaris*

The fruiting bodies of *C. militaris* were identified using micro and macromorphological analyses and confirmed at the Department of Biotechnology, Bodoland University, Assam, India, as per our earlier published reports [26]. Our reported method carried out solid-state cultivation, fruiting, and harvesting of mycelial culture. The harvested samples were frozen at −86 °C for 36 h before storing in an airtight container.

### 2.2 Preparation of *C. militaris* extract

The *C. militaris* powder (10 g) was placed in a 250 mL iodine flask, and 100 mL of double-distilled water was added. This mixture was then heated for 30 min in a water bath. The resulting concoction was filtered through the No. 1 Whatman filter paper. The resulting aqueous extract was placed in a suitable container and refrigerated at 4 °C until use [27].

### 2.3 Pharmacoinformatic analysis

#### 2.3.1 Molecular docking (MD)- ligand-protein docking

The glide module of the Schrodinger suite was used to dock the individual bioactive constituents into the identified binding site of the grid prepared using the Crystal structure of the SARS-CoV-2 spike receptor-binding domain bound with ACE2 (PDB: 6M0J). The binding site was selected based on the results from the SiteMap algorithm in the Schrodinger suit. The binding site was formed of following residues; Arg403, Tyr405, Arg408, Gly409, Glu453, Lys417, Ile418, Phe456, Tyr449, Gln498, Leu452, Gln493, Ser494, Gly447, Phe497, Asn501, Gly504 and Tyr505. The lowest binding pose of each docking run was chosen to visualize the ligand-protein interaction in glide XP pose visualizer. The crucial active site interactions were analyzed along with the scoring functions and further selected for Molecular dynamics simulation (MDS) [[28], [29], [30], [31]].

#### 2.3.2 MDS and molecular mechanics-generalized born solvent accessibility (MM-GBSA) analysis

The AMBER18 MDS package performed all the simulation calculations. All the ligand molecules were parameterized with the ANTECHAMBER tool implemented with AMBER18 and AMBER20 tools. The protein-ligand complexes were prepared in XLEAP to obtain an explicit solvent model with TIP3P water molecules. GAFF and FF14SB forcefields were used to model the ligand and protein, respectively. These complexes were subjected to minimization cycles, simulated annealing, equilibration, and a production run of 100 ns on an Nvidia V100-SXM2-16GB Graphic Processing Unit using the PMEMD.CUDA module. Further, the 100 ns trajectories were stripped of water molecules and salt ions with CPPTRAJ. The MM-GBSA analysis was performed on the solvated trajectories using Amber18 and Amber20 tools on all the 10000 frames [[32], [33], [34], [35]].

#### 2.3.3 Network pharmacology

The SMILES of bioactives were retrieved from PubChem [https://pubchem.ncbi.nlm.nih.gov/] database and queried in DIGEP-Pred [http://www.way2drug.com/ge/] database to identify the up-/down-regulated targets (proteins) at the pharmacological activity (Pa) 0.5. The list of regulated targets was then queried in the STRING [https://string-db.org/] database for enrichment analysis for cellular components, molecular function, and biological processes. Similarly, the regulated pathways were recorded regarding the KEGG [https://www.genome.jp/kegg/] pathway database. Similarly, a Java-based tool, i.e., Cytoscape [https://cytoscape.org/], was used to construct a network between bioactives, their targets, and regulated pathways. Possible duplicates were removed during the construction of the network and analyzed based on edge count [[36], [37], [38]].

### 2.4 Biological activities

#### 2.4.1 Cell line and virus

The Vero E6 cell line was procured from the National Centre for Cell Science, Pune, India, and was maintained in Dulbecco's modified Eagle's medium (DMEM) supplemented with 10% Fetal bovine serum (FBS) (PAN Biotech, Germany) and Penicillin-streptomycin. The SARS-CoV-2 clinical isolate (Acc.no. EPI_ISL_1196305) adapted in Vero E6 cells was used for this study [39].

#### 2.4.2 *Cytotoxicity* assay

The maximum non-cytotoxic (MNCT) concentration of *C. militaris* aqueous extract solubilized at 10 mg/mL concentration in DMSO was assayed by the EZcount™ MTT cell assay kit (Himedia, India) as per the manufacturer's protocol. Briefly, Vero E6 cells were seeded in a 96-well plate at approximately 10^4^ cells/well density. At 70% confluency, cells were treated with 25 μg/mL, 50 μg/mL, 75 μg/mL, and 100 μg/mL concentrations of the extract in triplicate along with DMSO as a reagent control. After 22 h, the wells were washed with 1X PBS, and subsequently, 10 μL MTT reagent (5 mg/mL) (Himedia, India) was added to the cells and incubated for 2–3 h at 37 °C. The formazan crystals formed were solubilized using 100 μL of the solubilization buffer at 37 °C for 15 min. The absorbance was measured at 570 nm using a multimode plate reader, and the metabolically active cell percentage was compared with the control cells to determine the cellular cytotoxicity [40].

#### 2.4.3 Antiviral assay

To assess the anti-SARS-CoV-2 activity of the *C. militaris* aqueous extract *in-vitro*, at 80% confluency, Vero E6 cells were infected in triplicate with SARS-CoV-2 at Multiplicity of Infection (MOI) 0.1 followed by 90 min incubation with shaking at every 10–15 min interval as described earlier [41]. After viral adsorption, the cells were washed with 1X sterile PBS and treated with different extract concentrations (25, 50, 75, 100 μg/mL) in complete DMEM. Remdesivir (100 μM) was taken as a positive control. The supernatants were extracted for RNA at 22 h post-infection.

#### 2.4.4 RNA extraction and qRT-PCR

The viral RNA was extracted from the cell culture supernatants using the TAN Bead Maelstrom 4800 automated RNA extraction platform, and cDNA was synthesized using the Prime Script First strand cDNA synthesis kit (Takara, Japan). The synthesized cDNA was subjected to the SYBR Green (Mesagreen SYBR Green-No ROX, Eurogentec, Belgium) based RT-qPCR targeting Nucleocapsid (N) gene for quantifying the viral RNA in cell culture supernatant [39,42].

## 3 Results and discussion

Previously published reports and *in-silico* studies [13,14,17,20,22,43] of the adenosine analogue of Cordycepin compelled us to scientifically validate the plausible potential of *C. militaris* in treating COVID-19 by *in-vitro* assays.

### 3.1 MD and MDS studies

**Fig. 1:**
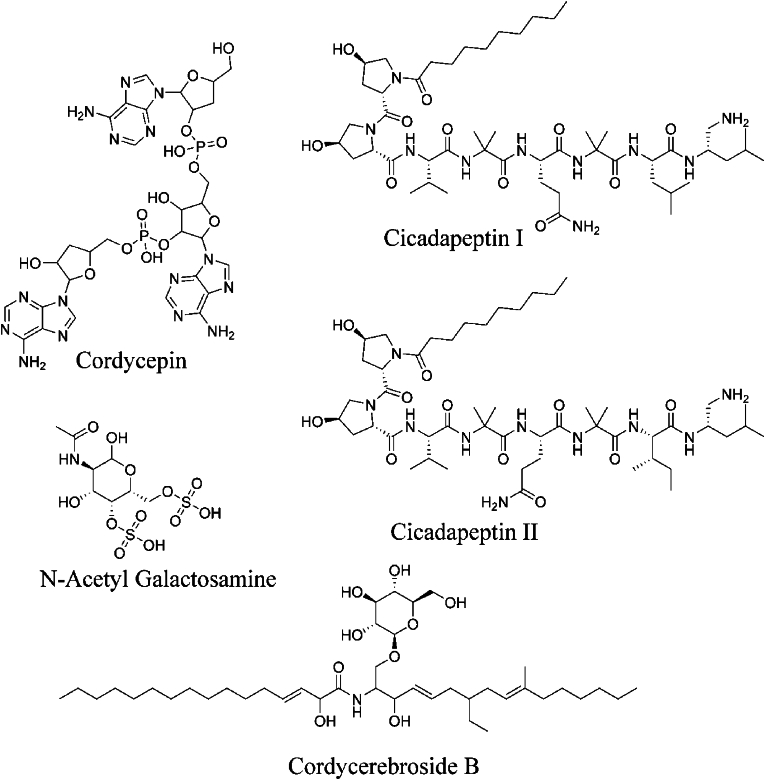
Structure of compounds found promising in the MD studies**.**

Forty-two constituents (Table S1) of the *C. militaris* were studied for MD studies. These ligands were docked in the receptor binding domain of the SARS-CoV-2 spike protein and ranked based on their highest binding affinity. The compounds Cordycepin, Cicadapeptin-I, Cicadapeptin-II, Cordycerebroside-B, and N-Acetyl galactosamine were found to be top scorers (Fig. 1). These compounds formed hydrogen bond interactions with the binding site residues (Table 1).

**Table 1:** Results from the MD studies of selected compounds on the interface site of SARS-CoV-2 spike RBD (PDB ID: 6M0J).

Sr. No.	Ligands	PubChem	Dock Score (kcal/mol)	H-Bonding	Other interactions
1	Cordycepin	452852	−9.718	Ser494, Glu484, Tyr449, Tyr453, Glu406	Tyr505, Arg403
2	Cicadapeptin I	11400450	−9.457	Glu484, Gln493, Gly496, Gln498, Try505, Arg403	–
3	Cicadapeptin II	11205390	−8.317	Gln493, Glu484, Ser494, Tyr453, Arg403, Glu406, Gln409	–
4	Cordycerebroside B	146683465	−7.26	Tyr453, Ser494, Glu406, Arg403, Gly496, Gln493	–
5	N-Acetyl galactosamine	22833549	−6.339	Ser494, Gly496, Tyr453, Arg403, Tyr505	–
6	Remdesivir	121304016	−5.94	Arg 403, Glu 406, Tyr 453	–

The top five ligands in the complex with the binding protein (Table 1) were further studied by MDS studies and binding free energy studies by MM-GBSA.

**Fig. 2:**
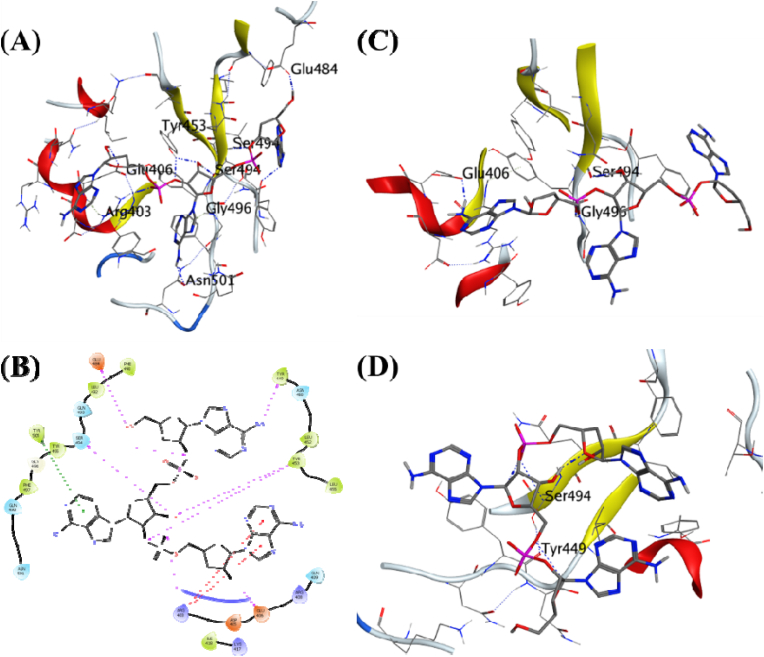
**Ligand-protein interactions**. **(A)** MD of Cordycepin on the interface of the SARS-CoV-2 receptor binding domain and ACE2 receptor. **(B)** Two-dimensional interaction map for the Cordycepin with the interface residues. **(C)** Initial frame of the production phase for the MDS of the docked ligand-receptor complex. **(D)** The final frame of the production phase for the 100 ns MDS of the docked ligand-receptor complex.

Cordycepin was predicted as the ligand with the highest binding energy of −9.71 kcal/mol in the MDS. It formed hydrogen bonds with the surrounding residues like Ser494, Glu484, Tyr449, Tyr453, and Glu406 (Fig. 2A). Cordycepin also formed π-π interaction with the Tyr505. MDS studies further studied this complex for its stability with the docked protein. The analysis of the complex through 100 ns trajectory showed hydrogen bond formation beginning from the simulations with Glu406, Ser494, and Gly496 (Fig. 2C).

**Fig. 3:**
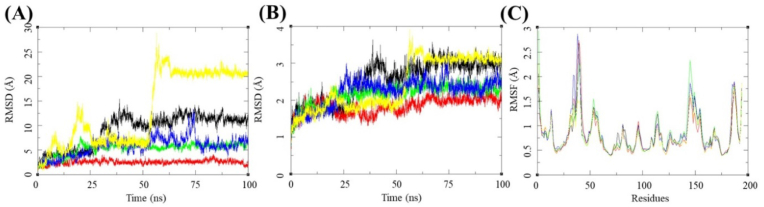
**MDS for the selected complexes from the MD experiments**. **(A)** Root mean square deviation (RMSD) of Cordycepin (Black), Cicadapeptin-I (Red), Cicadapeptin-II (Green), Cordycerebroside-B (Blue), N-Acetyl galactosamine (Yellow) bound to SARS-CoV-2 spike receptor-binding domain (PDB: 6M0J). **(B)** Root Mean Square Deviation (RMSD) of SARS-CoV-2 spike receptor-binding domain (PDB: 6M0J) residues, Cordycepin (Black), Cicadapeptin-I (Red), Cicadapeptin-II (Green), Cordycerebroside-B (Blue), and N-Acetyl galactosamine (Yellow). **(C)** Root mean square fluctuations (RMSF) of SARS-CoV-2 spike receptor-binding domain residues in complex with Cordycepin (Black), Cicadapeptin-I (Red), Cicadapeptin-II (Green), Cordycerebroside-B (Blue), and N-Acetyl galactosamine (Yellow).

Cordycepin is a large molecule with a molecular weight of 893.66 with multiple rotatable bonds, allowing it to achieve several conformations. The MDS RMSD for *Cordyceps* shows a gradual rise for an initial 30 ns till it stabilizes at 14 Å, and then the RMSD fluctuations stabilize between 2 and 3 Å for the rest of the MDS (Fig. 3A). The protein RMSD fluctuations are low and stay stable for most of the simulation (Fig. 3B). The RSMF per residue is low. It is between 0.5 and 1.5 Å, with some patches like aa30-40 and aa140-160 having slightly higher sides of the RMSF of 2.75 and 2.0 Å, respectively (Fig. 3C). This stability can be attributed to the hydrogen bonds between Ser494 and Tyr449 observed in the final frame of the MDS (Fig. 2B–D). The binding free energy was calculated for this complex by the MMGBSA method, and the Binding free energy (ΔGbind) was recorded as −7.06 (5.84) kcal/mol based on the whole trajectory analysis (Table 2).

**Table 2:** Binding free energy components for the protein-ligand complexes calculated by MM-GBSA analysis.

Compounds	MM-GBSA*						
	ΔE_VDW_	ΔE_ELE_	ΔG_GB_	ΔG_Surf_	ΔG_gas_	ΔG_Sol_	ΔG_bind_
*Cordyceps* derivatives							
Cordycepin	−31.20 (5.91)	34.64 (20.88)	−7.33 (19.48)	−4.21 (0.57)	4.48 (19.43)	−11.55 (19.43)	−7.06 (5.84)
Cicadapeptin-I	−54.89 (4.89)	−114.68 (10.40)	122.18 (8.43)	−7.24 (0.51)	−198.94 (11.15)	114.94 (8.31)	−84.00 (5.83)
Cicadapeptin-II	−43.66 (5.64)	−94.72 (39.31)	102.90 (35.79)	−6.24 (0.68)	−167.40 (39.53)	96.65 (35.74)	−70.75 (6.81)
Cordycerebroside-B	−33.21 (7.49)	−22.17 (16.19)	37.94 (16.27)	−4.99 (1.28)	−73.74 (21.39)	32.94 (15.15)	−40.79 (7.37)
N-Acetyl galactosamine	−12.75 (3.48)	−24.83 (11.76)	38.33 (11.69)	−2.38 (0.54)	−36.33 (13.80)	35.94 (11.32)	−0.38 (4.48)

**ΔE**_**VDW**_ = van der Waals contribution from MM; **ΔE**_**ELE**_ = electrostatic energy as calculated by the MM force field; **ΔG**_**GB**_ = the electrostatic contribution to the solvation-free energy calculated by GB; **ΔG**_**Surf**_ = solvent-accessible surface area; **ΔG**_**Sol**_ = solvation free energy; **ΔG**_**gas**_ = gas phase interaction energy; **ΔG**_**bind**_ = Binding free energy. All energies are in kcal/mol with standard deviation in parenthesis.

**Fig. 4:**
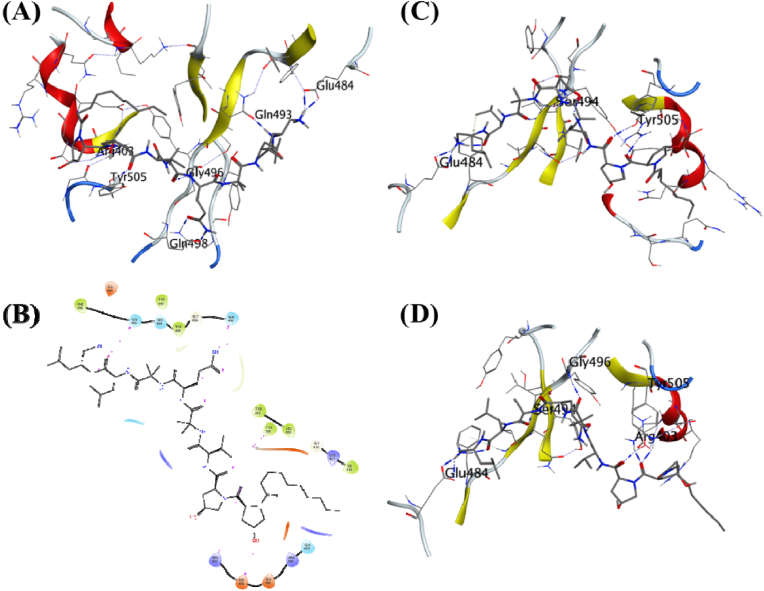
**Ligand-protein interactions**. **(A)** MD of Cicadapeptin-I on the SARS-CoV-2 receptor binding domain and ACE2 receptor interface. **(B)** Two-dimensional interaction map for the Cicadapeptin-I with the interface residues. **(C)** Initial frame of the production phase for the MDS of the docked ligand-receptor complex. **(D)** The final frame of the production phase for the 100 ns MDS of the docked ligand-receptor complex.

Cicadapeptin-I was predicted as the ligand with the second-highest binding energy of −9.45 kcal/mol. It also formed hydrogen bonds with the surrounding residues like Glu484, Gln493, Gly496, Gln498, Try505, and Arg403 (Fig. 4A). The MDS studies further studied Cicadapeptin-I in complex with the RBD for its stability with the docked protein.

The analysis of the complex through 100 ns trajectory shows several hydrogen bonds were present during the beginning of the simulations Glu484, Ser494, and Tyr505 (Fig. 4C). Cicadapeptin-I is a large molecule with a molecular weight of 1007.33, and multiple rotatable bonds and side chains allow it to achieve several conformations. However, the analysis of its MDS trajectory suggests it to be highly stable with ligand RMSD below 3 Å throughout the MDS (Fig. 3A), its protein RMSD is also low, about 2 Å (Fig. 3B), and the RMSF per residue falls on the lower side of the global comparison with the other complexes (Fig. 3C). The binding free energy (ΔGbind) was recorded as −84.00 (5.83) kcal/mol, the highest compared to all other complexes under investigation (Table 2). The visual analysis of the last frame of the simulation and the complete trajectory shows the presence of hydrogen bond interaction between Glu484, Ser494, Gly496, and Tyr505 is intact throughout the 100 ns trajectory (Fig. 4B–D). These observations suggest greater affinity and stability of the Cicadapeptin-I bound to the RBD of SARS-CoV-2 spike protein.

**Fig. 5:**
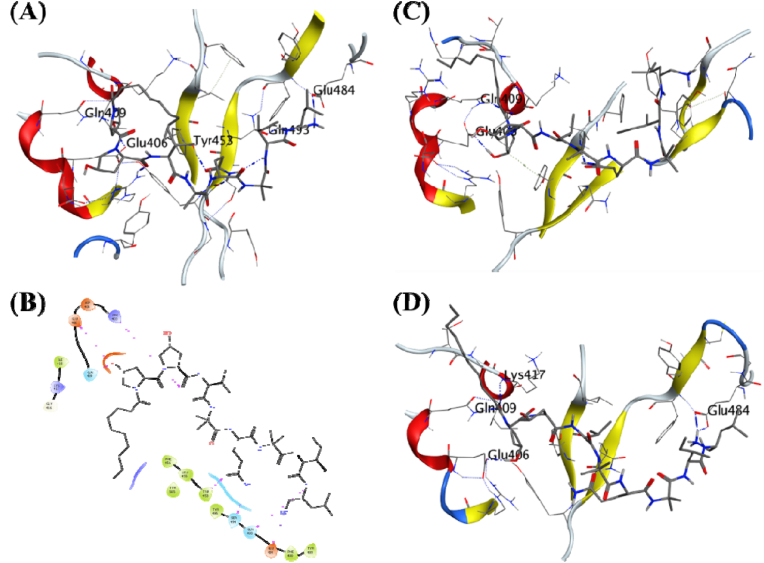
**Ligand-protein interactions**. **(A)** MD of Cicadapeptin-II on the SARS-CoV-2 receptor binding domain and ACE2 receptor interface. **(B)** Two-dimensional interaction map for the Cicadapeptin-II with the interface residues. **(C)** Initial frame of the production phase for the MDS of the docked ligand-receptor complex. **(D)** The final frame of the production phase for the 100 ns MDS of the docked ligand-receptor complex.

Cicadapeptin-II is a stereoisomer of Cicadapeptin-I, and it was predicted to be the ligand with the third-highest binding energy of −8.317 kcal/mol. It also formed hydrogen bonds with the surrounding residues like Gln493, Glu484, Ser494, Tyr453, Arg403, Glu406, and Gln409 (Fig. 5A, Table 1). The MDS studies further studied Cicadapeptin-II in complex with the RBD for its stability with the docked protein. The analysis of the complex through 100 ns trajectory shows several hydrogen bonds were present during the beginning of the simulations Glu408 and Gln409 (Fig. 5C). Cicadapeptin-II is also a large molecule with a molecular weight of 1007.33, and multiple rotatable bonds and side chains allow it to achieve several conformations. The analysis of its MDS trajectory suggests it to be highly stable with ligand RMSD below 5 Å throughout the MDS (Fig. 3A), its protein RMSD is also low, about 2.25 Å (Fig. 3B), and the RMSF per residue falls on the lower side of the global comparison with the other complexes (Fig. 3C). The binding free energy (ΔGbind) was recorded as −70.75 (6.81) kcal/mol and this was the second highest compared to other complexes under investigation (Table 2). The visual analysis of the last frame of the simulation and the complete trajectory shows the presence of hydrogen bond interaction between Glu406, Gln409, Lys417, and Glu484 was intact during the 100 ns trajectory (Fig. 5B–D). These observations suggest comparable affinity and stability of the Cicadapeptin-II with the Cicadapeptin-I bound to the RBD of SARS CoV-2 spike protein.

**Fig. 6:**
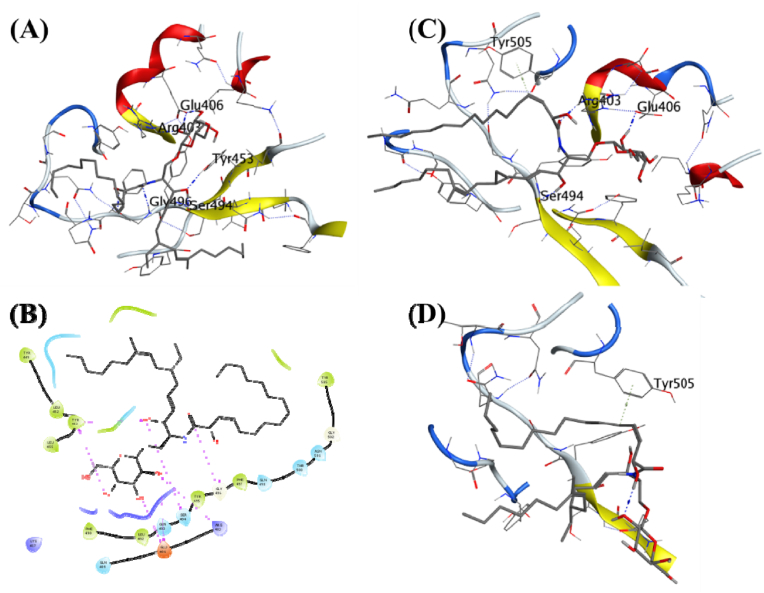
**Ligand-protein interactions**. **(A)** MD of Cordycerebroside-B on the SARS-CoV-2 receptor binding domain and ACE2 receptor interface. **(B)** Two-dimensional interaction map for the Cordycerebroside-B with the interface residues. **(C)** Initial frame of the production phase for the MDS of the docked ligand-receptor complex. **(D)** The final frame of the production phase for the 100 ns MDS of the docked ligand-receptor complex.

Cordycerebroside-B is a linear ligand with a molecular weight of 726.05 possessing a binding energy of −7.26 kcal/mol. It formed hydrogen bonds with the surrounding residues like Tyr453, Ser494, Glu406, Arg403, Gly496, and Gln493 (Fig. 6A). The MDS studies further studied cordycerebroside-B in complex with the RBD for its stability with the docked protein. The analysis of the complex through 100 ns trajectory shows several hydrogen bonds were present during the beginning of the simulations: Ser494, Arg403, Glu406, and Tyr505 (Fig. 6C). The analysis of its MDS trajectory suggests it to be less stable with ligand RMSD around 5 Å throughout the MDS with some larger fluctuations, its protein RMSD is about 2.25 Å, and the RMSF per residue falls on the lower side of the global comparison with the other complexes (Fig. 3A–C).

The binding free energy (ΔGbind) was recorded as −40.79 (7.37) kcal/mol. This is the third highest compared to all other complexes under investigation. The visual analysis of the last frame of the simulation and the complete trajectory shows the loss of hydrogen bond interaction between Glu406, Gln409, Lys417, and Glu484 and the ligand. A π-π interaction was formed with Tyr505 towards the end of the 100 ns trajectory (Fig. 6B–D). These observations suggest lower affinity and less stability of the Cordycerebroside-B compared with Cicadapeptin-I bound to the RBD of SARS CoV-2 spike protein.

**Fig. 7:**
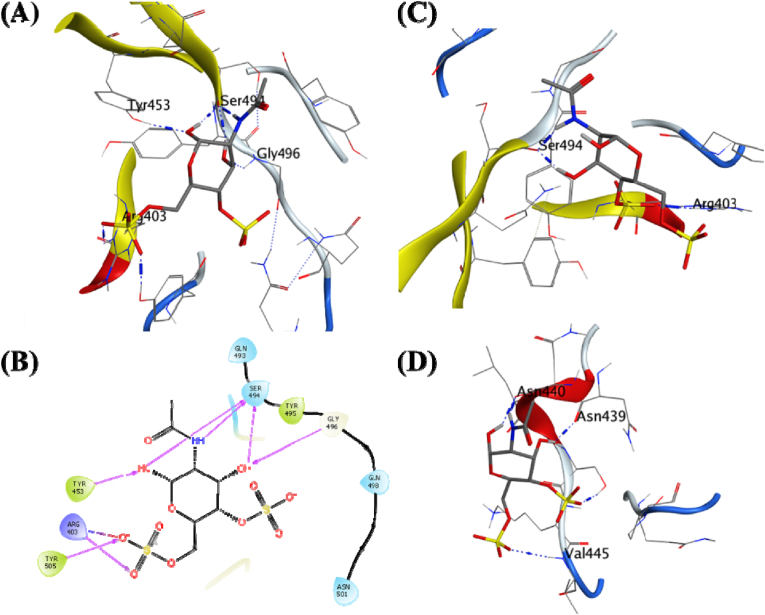
**Ligand-protein interactions**. (A) MD of N-Acetyl galactosamine on the SARS-CoV-2 receptor binding domain interface and ACE2 receptor. (B) Two-dimensional interaction map for the N-Acetyl galactosamine with the interface residues. (C) Initial frame of the production phase for the MDS of the docked ligand-receptor complex. (D) The final frame of the production phase for the 100 ns MDS of the docked ligand-receptor complex.

N-acetyl galactosamine is a small ligand with a molecular weight of 381.32 which was predicted to have a binding energy of −6.33 kcal/mol (Fig. 7A).

It formed hydrogen bonds with the surrounding residues like Ser494, Gly496, Tyr453, Arg403, and Tyr505 (Fig. 7C). The MDS studies further studied N-acetyl galactosamine in complex with the RBD for its stability with the docked protein. The analysis of the complex through 100 ns trajectory shows several hydrogen bonds were present during the beginning of the simulations Ser494 and Arg403. The analysis of its MDS trajectory suggests it is less stable, with several fluctuations in the ligand RMSD around 15 Å throughout the MDS with more significant fluctuations (Fig. 7A). The ligand RMSD stabilized after 50 ns with 2–4 Å, and its protein RMSD and RMSF for the protein residues were also higher than the rest of the complexes (Fig. 3B and C). The binding free energy (ΔGbind) was recorded as −0.38 (4.48) kcal/mol. This was the lowest compared to all other complexes under investigation, as is the reference Remdesivir (Table 2). The visual analysis of the last frame of the simulation and the complete trajectory showed the formation of some new hydrogen bond interaction between Asn440, Asn439, and Val445 and the ligand. The appearance of these bonds justifies the stable ligand RMSD towards the end of the 100 ns trajectory (Fig. 7B–D). These observations suggest lower affinity and less N-acetyl galactosamine stability than Cicadapeptin-I bound to the RBD of the SARS-CoV-2 spike protein.

### 3.2 Network pharmacology analysis

A total of 12 bioactives *viz*. adenosine, Cinnamic acid, Citric acid, Cordycepin, dipicolinic acid, Ergosterol, Fumaric acid, Hypoxanthine, N-acetylgalactosamine, *p*-hydroxybenzoic acid, β-Sitosterol, and δ-tocopherol were considered for network construction. Among them, *p*-hydroxybenzoic acid, fumaric acid, and cinnamic acid were identified to have the highest number of interactions. P-hydroxybenzoic acid was identified as the target of 31 proteins, fumaric acid targeted 30 proteins, and cinnamic acid targeted 27 proteins. Among the 31 proteins regulated by *p*-hydroxybenzoic acid, 13 were downregulated, and 18 were upregulated. Within these, a cluster of differentiation 86 exhibited the highest activity (Pa = 0.886) for upregulation, while peroxisome proliferator-activated receptor-α showed the maximum downregulated activity (Pa = 0.901). Furthermore, ten of the 30 proteins regulated by fumaric acid were downregulated, and 20 were upregulated. Among these, vimentin exhibited the highest activity (Pa = 0.819) for downregulation, while retinoic acid receptor-α showed the maximum activity for upregulation (Pa = 0.876). Similarly, within 30 proteins regulated by cinnamic acid, 10 experienced downregulation, while 17 underwent upregulation. Notably, vimentin displayed the highest activity (Pa = 0.737) for downregulation, whereas retinoic acid receptor-α demonstrated the maximum activity for upregulation (Pa = 0.927).

**Fig. 8:**
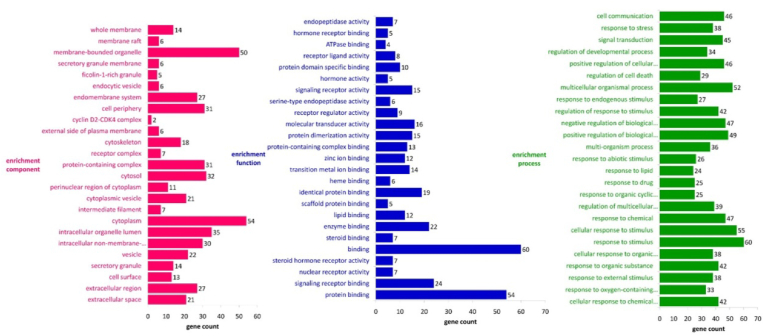
Functional enrichment analysis of protein-protein interaction regulated by biomolecules from *C. militaris*.

Biomolecules were identified to target the different cellular compartments, i.e., cell membrane, cytoplasm, cytoplasmic vesicle, cytoskeleton, and endomembrane system. In addition, this could affect other molecular functions like endopeptidase, ligand-receptor, hormone, signalling receptor, protein dimerization, and nuclear receptor activities followed by binding of ATPase, lipid, zinc ion, and proteins. This could further affect cellular communication, signal transduction, and response to stress-associated stimuli and chemicals. GO analysis identified a total of 57 GO terms for enrichment components in which extracellular space was highly enriched at the false discovery rate of 0.0000000117 by regulating 21 genes (TNFRSF1A, HMOX1, TIMP1, MMP2, PLAT, CCL2, COL1A1, MMP7, PTH, FLT1, LEP, GH1, KLK3, CLU, TAC1, HBA1, CD86, CTSB, PLAU, NPPB, ADIPOQ) against 1134 background gene count at the strength of 0.75 (Fig. 8). Further, among 51 molecular functions, the enrichment function identified the prime regulation of protein binding at the false discovery rate of 4.40E-14 by regulating 54 genes (TNFRSF1A, HMOX1, GSS, TIMP1, PLAT, CCL2, COL1A1, NR3C1, SELL, CAT, KRT1, RARA, CDK4, MDM2, CCND2, CYBA, PTH, FLT1, KRT17, LEP, GH1, CLU, TAC1, PGR, NOS2, CYP3A4, ESR2, CTSB, ATG7, RAC1, CASP8, TFRC, GYPA, IVL, AR, ABCA1, ID1, NPPB, SMN2, KRT18, NFE2L2, ELAVL1, PPARA, CHEK1, ADIPOQ, STRAP, CD36, GPX1, PRKCA, TOP2A, VIM, VDR, KRT8, VAV1) against 6607 background proteins at the strength of 0.4 (Fig. 8). Also, among 781 GO terms, the biological process identified the prime regulation of cellular response to the chemical stimulus at the false discovery rate of 6.65E-18 by regulating 42 genes (TNFRSF1A, HMOX1, TIMP1, MMP2, CCL2, COL1A1, NR3C1, CAT, RARA, CDK4, MDM2, CYBA, PTH, FLT1, CD14, LEP, GH1, TAC1, HBA1, PGR, NOS2, CD86, CYP3A4, ESR2, CTSB, ATG7, RAC1, CASP8, TFRC, AR, ABCA1, ID1, KRT18, NFE2L2, PPARA, ADIPOQ, CD36, GPX1, VIM, VDR, KRT8, VAV1) against 2672 background proteins at the strength of 0.68 (Fig. 8).

**Fig. 9:**
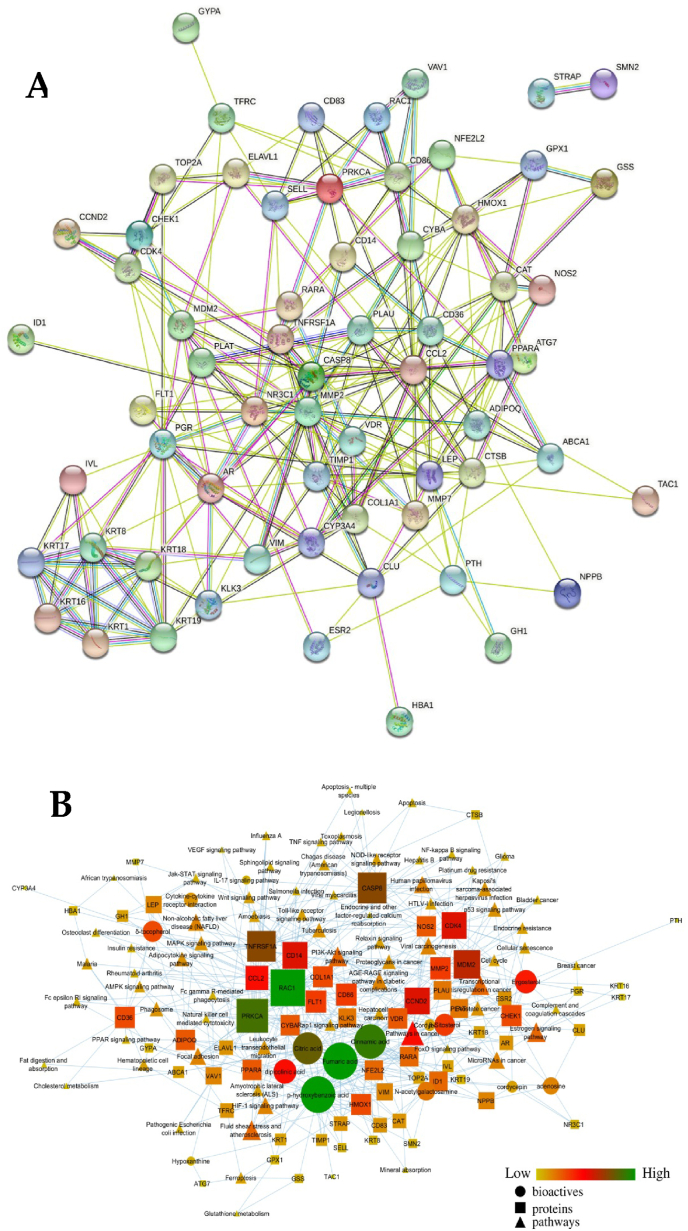
(a) Protein-protein interaction of the bioactive-targeted proteins. Node color; coloured nodes: query proteins and first shell of interactors; from curated databases, experimentally determined, Predicted Interactions; gene neighbourhood, gene fusions, gene co-occurrence and Others; text mining, co-expression, protein homology. (b) Compounds-proteins-pathways interaction. Circular, square and triangle nodes represent the compounds, proteins and pathways, respectively. The size of the node is directly proportional to the edge count.

Additionally, KEGG pathways analysis identified pathways in cancer to be primarily regulated at the false discovery rate of 2.40E-07 by regulating 14 genes (HMOX1, MMP2, RARA, CDK4, MDM2, CCND2, KLK3, NOS2, ESR2, RAC1, CASP8, AR, NFE2L2, PRKCA) against 515 background proteins at the strength of 0.68 (Table S2). Similarly, the protein interaction network of the bioactives regulated targets is illustrated (Fig. 9a) including the complete interaction of the bioactives, their regulated proteins, and modulated pathways (Fig. 9b).

In the present study, network pharmacology combined the approaches from experimental evidence followed by data mining in STRING, including the protein-protein interaction and complex network analysis between the interacting biomolecules, regulated proteins, and pathways. Herein, we identified the possible targets to be modulated and utilized the concept of polypharmacology, where the pool of active ingredients could have a multitargeting strategy and cellular compartments (e.g., vesicles and membrane), different molecular functions (e.g., binding and regulation of varying surface receptors) and biological spectra (e.g., stress response) were traced. In addition, the combined interaction of the phytochemicals identified the endogenous stimulus regulation linked with cytokine storm, Negative instruction of Toll-Like Receptors, Apoptosis of Infected Cells, negative feedback loops, and regulation of Interferon signalling. In addition, the coronavirus targets the cell membrane (ACE2 protein) to invade the cell. In the present study, we traced this combined interaction to counteract this process, evidenced by the tracing of membrane genes in functional enrichment analysis.

**Fig. 10:**
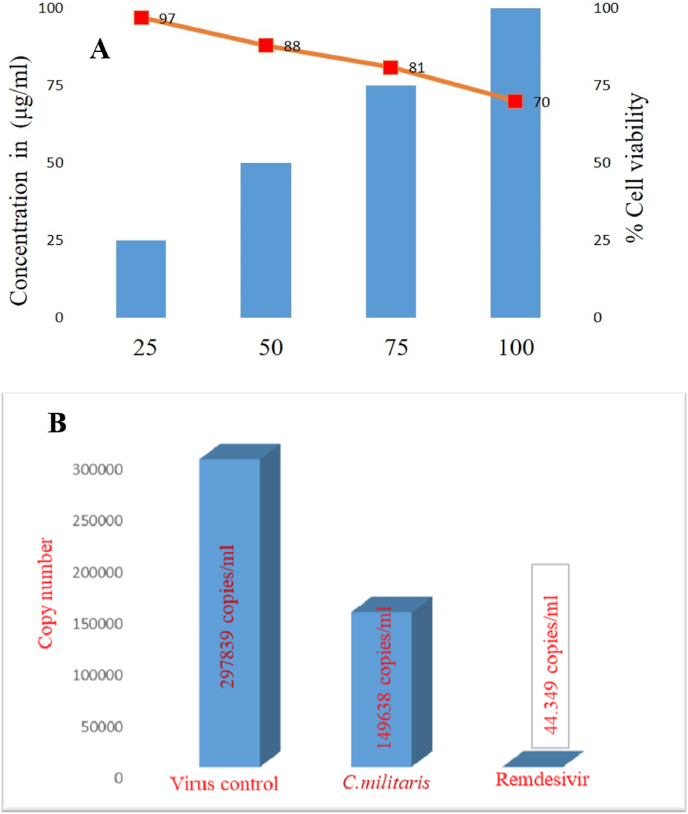
(**a)** MTT cytotoxicity assay presenting the cytotoxicity of *C. militaris* on Vero E6 cell lines. The column represents the concentration (μg/mL) of *C. militaris,* and the curve denotes the cell viability (%) at that concentration. **(b)** Anti-SARS-SoV-2 activity of crude extract of *C. militaris* compared to the Remdesivir in Vero E6 cell line. The column denotes the number of viral copies number per mL.

Notably, the viral copy numbers were reduced by 50.24% even when using crude extract at 100 μg/mL concentration Fig. 10a. The Cordycepin in pure or its analog has been shown to have significant anti-SARS-CoV-2 activity Fig. 10b [13,15]. The extraction method and the form in which *C. militaris* was assayed might have resulted in the variation in the activity. Moreover, the main active ingredient, Cordycepin, has clinically sustained action against COVID-19 and high metabolic stability [13]. Furthermore, the network pharmacological analysis of Cordycepin revealed ADAM17 as a potential target for anti-COVID-19 therapy and might be of medical significance for cancer patients infected with SARS-CoV-2 [42].

Also, the extract of *C. militaris* has been shown to be effective against the 2009 pandemic H1N1 virus [44], Dengue [45], HIV-1, and Hepatitis Virus [46]. Besides antiviral properties, the polysaccharides of *C. militaris*, CMP40, and CMP50, have been found effective in significantly improving the immune effect of the Newcastle disease vaccine *in-vitro* and *in-vivo* [47,48]. The findings of this study may further be substantiated with more *in-vitro* studies using different variants of SARS-CoV-2 and *in-vivo* in animal models for effectiveness as a therapeutic agent in the fight against reemerging COVID-19 surge and endemicity. However, in the present network study, the focus was on regulated targets. It would be interesting to evaluate the network of binding proteins based on the Tanimoto index of established molecules, although some attempt was made through MDS. In addition, future studies are warranted to elucidate bioactive molecules with antiviral activity, their mechanisms of action, and their potential as an effective antiviral to combat reemerging SARS-CoV-2 or any future viral pandemic.

## 4 Conclusions

The MD analysis revealed that the Cordycepin has the highest binding affinity to the SARS-CoV-2 spike protein. Cicadapeptin-I, Cicadapeptin-II, Cordycerebroside-B, and N-Acetyl galactosamine have the highest binding affinity to the SARS-CoV-2 spike protein. The MD analysis of *C. militaris* revealed that the Cordycepin had the highest affinity (9.71 kcal/mol) than other bioactives. Cicadapeptin-I, Cicadapeptin-II, Cordycerebroside-B, and N-Acetyl galactosamine to the receptor binding domain of the SARS-CoV-2 spike protein. The crude aqueous extract of *C. militaris* did not affect the Vero E6 cell viability and retained more than 70% viability at 100 μg/mL concentration. The *C. militaris* was found to be active against SARS-CoV-2 as even crude aqueous extract (100 μg/mL) could reduce the viral copy numbers to 149638 copies/mL from 297839 copies/mL compared to the virus-only control, equating to a 50.24% reduction in viral particles. The research results and our findings reported in this study are preliminary findings and are in a state of continuous improvement.

## Data availability statement

Data will be made available on request.

## Funding sources

This research did not receive any specific grant from funding agencies in the public, commercial, or not-for-profit sectors.

## Declaration of generative AI in scientific writing

NIL.

## Author Contributions

SG and SD: Conceptualization, Methodology, Validation, Investigation, Data curation, Writing – review & editing. PG, RC, and PK: Methodology, Investigation, Validation, Writing – original draft. VB, RA, MA, and NG: Formal analysis, Resources, Validation, Drafting of MS, Writing – review & editing. MSK and RC: Funding acquisition, Formal analysis, drafting of MS, Writing – review & editing.

## Conflict of Interest

Dr SG is a part of JAIM's editorial board and was not involved in any manuscript review or editorial processes. Other authors declare that they have no competing financial interests or personal relationships that could have appeared to influence the work reported in this paper.
