# Supplementary material for: Quest for Anti-SARS-CoV-2 antiviral therapeutics: in-silico and in-vitro analysis of edible mushroom- Cordyceps militaris
Source: J Ayurveda Integr Med. 2024 Jun 12;15(3):100979. doi: 10.1016/j.jaim.2024.100979 (PMC11282376; doi:10.1016/j.jaim.2024.100979)
Supplement: Multimedia component 1 [file mmc1.doc]

**SUPPLEMENTARY FILE**

QUEST FOR ANTI-SARS-COV-2 ANTIVIRAL THERAPEUTICS: *IN-SILICO* AND *IN-VITRO* ANALYSIS OF EDIBLE MUSHROOM- *CORDYCEPS MILITARIS*

QUEST FOR ANTI-SARS-COV-2 ANTIVIRAL THERAPEUTICS: *IN-SILICO* AND *IN-VITRO* ANALYSIS OF EDIBLE MUSHROOM- *CORDYCEPS MILITARIS*

Pradeep Gandhale^1†^, Rupesh Chikhale^2†^, Pukar Khanal^3^, Vashkar Biswa^4^, Raju Ali^4^, Mohd Shahnawaz Khan^5^, Nilambari Gurav^6^, Muniappan Ayyanar^7^, Sandeep Das^4^*, Shailendra Gurav^8^*

*^1^ ICAR-National Institute of High-Security Animal Diseases, Bhopal, MP, India.*

*^2^ UCL School of Pharmacy, 29−39 Brunswick Square, London WC1N 1AX, UK*

*^3^ Department of Pharmacology, KLE College of Pharmacy Belagavi, KLE Academy of Higher Education and*

*Research (KAHER) Belagavi- 590010, India*

*^4^ Department of Biotechnology, Bodoland University, Assam, 783 390, India*

*^5^ Department of Biochemistry, College of Science, King Saud University, Riyadh, Saudi Arabia*

*^6^ Department of Pharmacognosy, PES’s Rajaram and Tarabai Bandekar College of Pharmacy, Ponda, Goa, India*

*^7^ Department of Botany, A.V.V.M. Sri Pushpam College (Autonomous), Poondi (Affiliated to Bharathidasan University), Thanjavur, Tamil Nadu, India*

*^8^ Department of Pharmacognosy, Goa College of Pharmacy, Goa University, Goa, India*

^†^ Equal contribution

*****Correspondence:

Dr Shailendra Gurav, [shailendra.gurav@nic.in](mailto:shailendra.gurav@nic.in);

Dr Sandeep Das, [sandeep_dna2003@yahoo.co.in](mailto:sandeep_dna2003@yahoo.co.in)

**Table S1:** Bioactives of *C. militrais* with their docking score

| **Sr. No.** | **Name of Bioactive** | **PUBCHEM ID** | **Docking Score** |
| --- | --- | --- | --- |
| 01 | Arachidic acid | 10467 | -2.016 |
| 02 | Behenic acid | 87508015 | -1.359 |
| 03 | Capric acid | 454075 | -0.983 |
| 04 | Caproic acid | 454079 | -1.473 |
| 05 | Caprylic acid | 379 | -2.43 |
| 06 | Cephalosporolide C | 44575720 | -4.795 |
| 07 | Cephalosporolide F | 42599539 | -3.268 |
| 08 | Chitinase | 92044409 | -4.266 |
| 09 | Cicadapeptin I | 11400450 | -9.457 |
| 10 | Cicadapeptin II | 11205390 | -8.317 |
| 11 | Cinnamic acid | 444539 | -2.607 |
| 12 | cis-11,14,17-Eicosatrienoic acid | 90470400 | -1.821 |
| 13 | cis-11,14-Eicosadienoic acid | 6439848 | -1.482 |
| 14 | cis-11-Eicosenoic acid | 5282768 | -1.995 |
| 15 | cis-5,8,11,14,17-Eicosapentaenoic acid | 446284 | -3.503 |
| 16 | Citric acid | 311 | -4.42 |
| 17 | Cordycepic acid | 6251 | -5.947 |
| 18 | Cordycepin | 452852 | -9.718 |
| 19 | Cordycerebroside B | 1.47E+08 | -7.26 |
| 20 | Cordyheptapeptide A | 11542476 | -4.704 |
| 21 | Dipicolinic acid | 10367 | -2.958 |
| 22 | Dipicolinic acid | 10367 | -1.511 |
| 23 | Ergosterol | 444679 | -3.363 |
| 24 | Erucic acid | 5281116 | -2.33 |
| 25 | Fumaric acid | 444972 | -2.449 |
| 26 | Heneicosanoic acid | 16898 | -1.258 |
| 27 | Heptadecanoic acid | 10465 | -1.065 |
| 28 | Hypoxanthine | 1.35E+08 | -2.945 |
| 29 | Lauric acid | 3893 | -0.23 |
| 30 | Lignoceric acid | 11197 | -1.359 |
| 31 | linoleic acid | 5280450 | -0.014 |
| 32 | Linolenic acid | 5280934 | -1.459 |
| 33 | Myriocin | 6438394 | -5.531 |
| 34 | Myristic acid | 11005 | -0.287 |
| 35 | Myristoleic acid | 5281119 | -0.893 |
| 36 | N-acetylgalactosamine | 22833549 | -6.339 |
| 37 | Nervonic acid | 5281120 | 0.24 |
| 38 | Oleic acid | 445639 | -1.192 |
| 39 | Ophiocordin | 5490846 | -5.76 |
| 40 | Palmitoleic acid | 445638 | -0.963 |
| 41 | p-hydroxybenzoic acid | 135 | -3.441 |
| 42 | Soyacerebroside I | 11104507 | -5.629 |
| 43 | Tricosanoic acid | 17085 | -0.78 |

**Table S2**: KEGG analysis of regulated pathways

| #term ID | term description | observed /background gene count | false discovery rate | matching proteins in network (labels) |
| --- | --- | --- | --- | --- |
| hsa05200 | Pathways in cancer | 14/515 | 2.40E-07 | HMOX1, MMP2, RARA, CDK4, MDM2, CCND2, KLK3, NOS2, ESR2, RAC1, CASP8, AR, NFE2L2, PRKCA |
| hsa04915 | Estrogen signalling pathway | 8/133 | 1.76E-06 | MMP2, RARA, KRT16, KRT17, PGR, ESR2, KRT19, KRT18 |
| hsa05418 | Fluid shear stress and atherosclerosis | 8/133 | 1.76E-06 | TNFRSF1A, HMOX1, MMP2, PLAT, CCL2, CYBA, RAC1, NFE2L2 |
| hsa05202 | Transcriptional misregulation in cancer | 8/169 | 5.15E-06 | PLAT, RARA, MDM2, CCND2, FLT1, CD14, CD86, PLAU |
| hsa04066 | HIF-1 signalling pathway | 6/98 | 4.29E-05 | HMOX1, TIMP1, FLT1, NOS2, TFRC, PRKCA |
| hsa04933 | AGE-RAGE signalling pathway in diabetic complications | 6/98 | 4.29E-05 | MMP2, CCL2, COL1A1, CDK4, RAC1, PRKCA |
| hsa04115 | p53 signalling pathway | 5/68 | 0.00011 | CDK4, MDM2, CCND2, CASP8, CHEK1 |
| hsa04920 | Adipocytokine signalling pathway | 5/69 | 0.00011 | TNFRSF1A, LEP, PPARA, ADIPOQ, CD36 |
| hsa04216 | Ferroptosis | 4/40 | 0.00024 | HMOX1, GSS, ATG7, TFRC |
| hsa04932 | Non-alcoholic fatty liver disease (NAFLD) | 6/149 | 0.00024 | TNFRSF1A, LEP, RAC1, CASP8, PPARA, ADIPOQ |
| hsa05206 | MicroRNAs in cancer | 6/149 | 0.00024 | HMOX1, MDM2, CCND2, PLAU, PRKCA, VIM |
| hsa04151 | PI3K-Akt signalling pathway | 8/348 | 0.00032 | COL1A1, CDK4, MDM2, CCND2, FLT1, GH1, RAC1, PRKCA |
| hsa05144 | Malaria | 4/47 | 0.00032 | CCL2, HBA1, GYPA, CD36 |
| hsa05215 | Prostate cancer | 5/97 | 0.00032 | PLAT, MDM2, KLK3, PLAU, AR |
| hsa05014 | Amyotrophic lateral sclerosis (ALS) | 4/50 | 0.00037 | TNFRSF1A, CAT, RAC1, GPX1 |
| hsa05203 | Viral carcinogenesis | 6/183 | 0.00042 | CDK4, MDM2, CCND2, RAC1, CASP8, CHEK1 |
| hsa04670 | Leukocyte transendothelial migration | 5/112 | 0.00045 | MMP2, CYBA, RAC1, PRKCA, VAV1 |
| hsa04510 | Focal adhesion | 6/197 | 0.00055 | COL1A1, CCND2, FLT1, RAC1, PRKCA, VAV1 |
| hsa04145 | Phagosome | 5/145 | 0.0013 | CYBA, CD14, RAC1, TFRC, CD36 |
| hsa01522 | Endocrine resistance | 4/95 | 0.0027 | MMP2, CDK4, MDM2, ESR2 |
| hsa04640 | Hematopoietic cell lineage | 4/94 | 0.0027 | CD14, TFRC, GYPA, CD36 |
| hsa05146 | Amoebiasis | 4/94 | 0.0027 | COL1A1, CD14, NOS2, PRKCA |
| hsa05152 | Tuberculosis | 5/172 | 0.0027 | TNFRSF1A, CD14, NOS2, CASP8, VDR |
| hsa04620 | Toll-like receptor signaling pathway | 4/102 | 0.0029 | CD14, CD86, RAC1, CASP8 |
| hsa05142 | Chagas disease (American trypanosomiasis) | 4/101 | 0.0029 | TNFRSF1A, CCL2, NOS2, CASP8 |
| hsa05167 | Kaposi's sarcoma-associated herpesvirus infection | 5/183 | 0.0029 | TNFRSF1A, CDK4, CD86, RAC1, CASP8 |
| hsa05219 | Bladder cancer | 3/41 | 0.0029 | MMP2, CDK4, MDM2 |
| hsa05205 | Proteoglycans in cancer | 5/195 | 0.0034 | MMP2, MDM2, RAC1, PLAU, PRKCA |
| hsa05165 | Human papillomavirus infection | 6/317 | 0.0042 | TNFRSF1A, COL1A1, CDK4, MDM2, CCND2, CASP8 |
| hsa04152 | AMPK signalling pathway | 4/120 | 0.0046 | LEP, ELAVL1, ADIPOQ, CD36 |
| hsa04110 | Cell cycle | 4/123 | 0.0049 | CDK4, MDM2, CCND2, CHEK1 |
| hsa05130 | Pathogenic Escherichia coli infection | 3/53 | 0.0049 | CD14, KRT18, PRKCA |
| hsa04926 | Relaxin signalling pathway | 4/130 | 0.0055 | MMP2, COL1A1, NOS2, PRKCA |
| hsa05416 | Viral myocarditis | 3/56 | 0.0055 | CD86, RAC1, CASP8 |
| hsa04310 | Wnt signalling pathway | 4/143 | 0.0074 | MMP7, CCND2, RAC1, PRKCA |
| hsa04664 | Fc epsilon RI signalling pathway | 3/67 | 0.0083 | RAC1, PRKCA, VAV1 |
| hsa05214 | Glioma | 3/68 | 0.0084 | CDK4, MDM2, PRKCA |
| hsa01524 | Platinum drug resistance | 3/70 | 0.0088 | MDM2, CASP8, TOP2A |
| hsa03320 | PPAR signalling pathway | 3/72 | 0.0089 | PPARA, ADIPOQ, CD36 |
| hsa04060 | Cytokine-cytokine receptor interaction | 5/263 | 0.0089 | TNFRSF1A, CCL2, FLT1, LEP, GH1 |
| hsa04218 | Cellular senescence | 4/156 | 0.0089 | CDK4, MDM2, CCND2, CHEK1 |
| hsa05225 | Hepatocellular carcinoma | 4/163 | 0.0099 | HMOX1, CDK4, NFE2L2, PRKCA |
| hsa04610 | Complement and coagulation cascades | 3/78 | 0.0103 | PLAT, CLU, PLAU |
| hsa04621 | NOD-like receptor signalling pathway | 4/166 | 0.0103 | CCL2, CYBA, CTSB, CASP8 |
| hsa04010 | MAPK signalling pathway | 5/293 | 0.0122 | TNFRSF1A, FLT1, CD14, RAC1, PRKCA |
| hsa05132 | Salmonella infection | 3/84 | 0.0122 | CD14, NOS2, RAC1 |
| hsa05323 | Rheumatoid arthritis | 3/84 | 0.0122 | CCL2, FLT1, CD86 |
| hsa04666 | Fc gamma R-mediated phagocytosis | 3/89 | 0.0135 | RAC1, PRKCA, VAV1 |
| hsa04657 | IL-17 signalling pathway | 3/92 | 0.0145 | CCL2, CASP8, ELAVL1 |
| hsa04064 | NF-kappa B signalling pathway | 3/93 | 0.0146 | TNFRSF1A, CD14, PLAU |
| hsa04015 | Rap1 signalling pathway | 4/203 | 0.0175 | FLT1, RAC1, ID1, PRKCA |
| hsa04215 | Apoptosis - multiple species | 2/31 | 0.0191 | TNFRSF1A, CASP8 |
| hsa04931 | Insulin resistance | 3/107 | 0.0202 | TNFRSF1A, PPARA, CD36 |
| hsa04668 | TNF signalling pathway | 3/108 | 0.0203 | TNFRSF1A, CCL2, CASP8 |
| hsa05145 | Toxoplasmosis | 3/109 | 0.0204 | TNFRSF1A, NOS2, CASP8 |
| hsa05143 | African trypanosomiasis | 2/34 | 0.021 | HBA1, PRKCA |
| hsa04071 | Sphingolipid signalling pathway | 3/116 | 0.0233 | TNFRSF1A, RAC1, PRKCA |
| hsa04975 | Fat digestion and absorption | 2/39 | 0.0262 | ABCA1, CD36 |
| hsa04380 | Osteoclast differentiation | 3/124 | 0.0269 | TNFRSF1A, CYBA, RAC1 |
| hsa04650 | Natural killer cell-mediated cytotoxicity | 3/124 | 0.0269 | RAC1, PRKCA, VAV1 |
| hsa04068 | FoxO signaling pathway | 3/130 | 0.0295 | CAT, MDM2, CCND2 |
| hsa05166 | HTLV-I infection | 4/250 | 0.0295 | TNFRSF1A, CDK4, CCND2, CHEK1 |
| hsa04210 | Apoptosis | 3/135 | 0.0316 | TNFRSF1A, CTSB, CASP8 |
| hsa04961 | Endocrine and other factor-regulated calcium reabsorption | 2/47 | 0.0335 | PRKCA, VDR |
| hsa04979 | Cholesterol metabolism | 2/48 | 0.0342 | ABCA1, CD36 |
| hsa05161 | Hepatitis B | 3/142 | 0.0345 | CDK4, CASP8, PRKCA |
| hsa00480 | Glutathione metabolism | 2/50 | 0.0358 | GSS, GPX1 |
| hsa04978 | Mineral absorption | 2/51 | 0.0366 | HMOX1, VDR |
| hsa05224 | Breast cancer | 3/147 | 0.0366 | CDK4, PGR, ESR2 |
| hsa05134 | Legionellosis | 2/54 | 0.0395 | CD14, CASP8 |
| hsa04630 | Jak-STAT signalling pathway | 3/160 | 0.0438 | CCND2, LEP, GH1 |
| hsa04370 | VEGF signalling pathway | 2/59 | 0.0451 | RAC1, PRKCA |
| hsa05164 | Influenza A | 3/168 | 0.0483 | TNFRSF1A, CCL2, PRKCA |

**Table S3:** Targets and regulatory bioactives counts

| **Targets** | **Related bioactives count** | **Targets** | **Related bioactives count** |
| --- | --- | --- | --- |
| ABCA1 | 2 | KLK3 | 5 |
| ADIPOQ | 1 | KRT1 | 4 |
| ADIPOQ | 4 | KRT16 | 1 |
| AR | 3 | KRT17 | 1 |
| ATG7 | 1 | KRT18 | 2 |
| CASP8 | 3 | KRT19 | 2 |
| CAT | 3 | KRT8 | 3 |
| CCL2 | 4 | LEP | 1 |
| CCND2 | 3 | MDM2 | 3 |
| CD14 | 6 | MMP2 | 2 |
| CD36 | 1 | MMP7 | 1 |
| CD83 | 5 | NFE2L2 | 7 |
| CD86 | 6 | NOS2 | 1 |
| CDK4 | 1 | NPPB | 5 |
| CHEK1 | 4 | NR3C1 | 2 |
| CLU | 3 | PGR | 1 |
| COL1A1 | 4 | PLAT | 3 |
| CTSB | 1 | PLAU | 1 |
| CYBA | 4 | PPARA | 5 |
| CYP3A4 | 1 | PRKCA | 1 |
| ELAVL1 | 3 | PTH | 1 |
| ESR2 | 1 | RAC1 | 6 |
| FLT1 | 3 | RARA | 5 |
| GH1 | 1 | SELL | 3 |
| GPX1 | 1 | SMN2 | 3 |
| GSS | 1 | STRAP | 5 |
| GYPA | 1 | TAC1 | 1 |
| HBA1 | 1 | TFRC | 1 |
| HMOX1 | 3 | TIMP1 | 3 |
| ID1 | 6 | TNFRSF1A | 2 |
| IVL | 4 | TOP2A | 3 |
| VDR | 6 | VAV1 | 1 |
| VIM | 5 |  |  |
